# Viscoelastic Particle Focusing and Separation in a Spiral Channel

**DOI:** 10.3390/mi13030361

**Published:** 2022-02-25

**Authors:** Haidong Feng, Alexander R. Jafek, Bonan Wang, Hayden Brady, Jules J. Magda, Bruce K. Gale

**Affiliations:** 1 Department of Mechanical Engineering, University of Utah, Salt Lake City, UT 84112, USA; haidong.feng@utah.edu (H.F.); u1058324@utah.edu (A.R.J.); u0949394@utah.edu (H.B.); 2 Department of Chemical Engineering, University of Utah, Salt Lake City, UT 84112, USA; bonan.wang@utah.edu (B.W.); magda@chemeng.utah.edu (J.J.M.)

**Keywords:** particle separation, viscoelastic flow, inertial focusing, spiral channel

## Abstract

As one type of non-Newtonian fluid, viscoelastic fluids exhibit unique properties that contribute to particle lateral migration in confined microfluidic channels, leading to opportunities for particle manipulation and separation. In this paper, particle focusing in viscoelastic flow is studied in a wide range of polyethylene glycol (PEO) concentrations in aqueous solutions. Polystyrene beads with diameters from 3 to 20 μm are tested, and the variation of particle focusing position is explained by the coeffects of inertial flow, viscoelastic flow, and Dean flow. We showed that particle focusing position can be predicted by analyzing the force balance in the microchannel, and that particle separation resolution can be improved in viscoelastic flows.

## 1. Introduction

Many biological and sensing assays require the ability to isolate particular types of particles from a mixture of particle types [1]. Existing label-free protocols select unique particle populations by relying on differences in particle size, shape, density, or surface properties [2]. While the optimal separation technique varies by target particle and application, generally the best separation method is one that is fast, robust, has a high throughput, and does not apply excessive force to the particles [3].

The field of microfluidics has been particularly well suited for applications involving particle separation and isolation [4,5]. Microfluidic devices manipulate fluids through channels with dimensions between 1–100 µm [6]. At this scale, channel features are commensurate with cells and physical phenomena arise which are unique to the microscale. In a microfluidic channel, particles can experience different types of forces that contribute to particle movement, retention, and separation. Force fields, such as electrical fields [7,8,9], surface acoustic waves [10], and optical forces [11], have been applied for particle manipulation and trapping in microfluidic channels. Furthermore, particles can experience forces with controllable magnitude and direction in a dielectrophoretic force field based on their size and polarizability, leading to particle separation with high resolution [12,13,14]. In optical trapping devices, particles experience the force due to incident photon scattering, and single particle manipulation is realized using optical forces [11]. One particularly useful hydrodynamic force for particle selection and separation on the microscale is inertial focusing [15,16,17]. Inertial focusing refers to the tendency of identical particles to align themselves to certain locations within a channel cross-section as they travel along a channel. Since the forces which drive this behavior are dependent on the particle geometry, it is possible to separate particles of different shapes or sizes in microfluidic channels [18,19]. In many applications, the efficiency of this separation can be increased through the additional incorporation of a Dean flow. In curved channels, a Dean flow arises due to the radial component of the fluid velocity and is generally expressed as counter-rotating vortices in the direction tangent to the fluid flow [20,21]. The Dean flow in the channel cross-section provides extra drag force leading to lateral particle movement. The inertial focusing force drives particles towards the channel top and bottom wall, while the Dean flow transports particle focusing positions to the channel inner sidewall. In inertial-Dean coupled flow, size-based separation can be realized since large particles have a focusing position closer to the inner sidewall when compared with small particles [22]. In addition, Dean flow can be induced in microfluidic channels with expansion/contraction structure [23] or pillar structures [24]. The Dean flow vortices are combined with inertial flow, and leads to a change of particle focusing position and particle trapping in the channel. A detailed description of inertial flow and Dean flow can be found in recent published review articles [25,26,27].

Viscoelastic fluid presents one type of non-Newtonian solution that exhibits both viscous and elastic properties under shear stress. In viscoelastic dominated flows, particle lateral movement and focusing stream phenomena have been observed and used for particle manipulation and separation in microchannels [28]. Polymer solutions, such as high molecular weight poly(ethylene) oxide (PEO) solutions, exhibit non-linear elastic stress distribution in the channel cross-section, leading to particles focusing towards the channel center [29,30]. In a confined channel, the particle focusing position is affected by the force balance between the viscoelastic forces and inertial forces [31]. In a straight channel, the interaction of the inertial focusing force and the viscoelastic focusing force leads to a change of particle focusing stream location. It is found that large particles bear split focusing streams near the channel sidewalls, while small particles focus to the channel center [32]. In a curved channel, the Dean flow leads to a change of particle focusing positions towards the channel outer sidewall, which effect has been applied for size-based particle separation [33].

In this paper, we demonstrate a particle separation regime showing the co-effects of inertial flow, viscoelastic flow, and Dean flow. With an increase of PEO concentration in the fluid, the force balance between the inertial flow and viscoelastic flow changes, leading to a change of particle focusing positions for different sized particles. The Dean flow, which provide a transverse drag force, improves the separation efficiency by changing particle focusing position in the horizontal direction. As we explore this separation regime, we principally focus on three main areas: (1) Contributions of the PEO solution viscoelastic properties under ultra-high shear rates in a microfluidic channel; (2) Identification of particle focusing positions and focusing regimes over a wide range of PEO concentrations; and (3) Evaluation and improvement of particle separation resolution in the viscoelastic flow. We expect that these results will find application in both sensing and biological assays.

## 2. Theory

**Figure 1:**
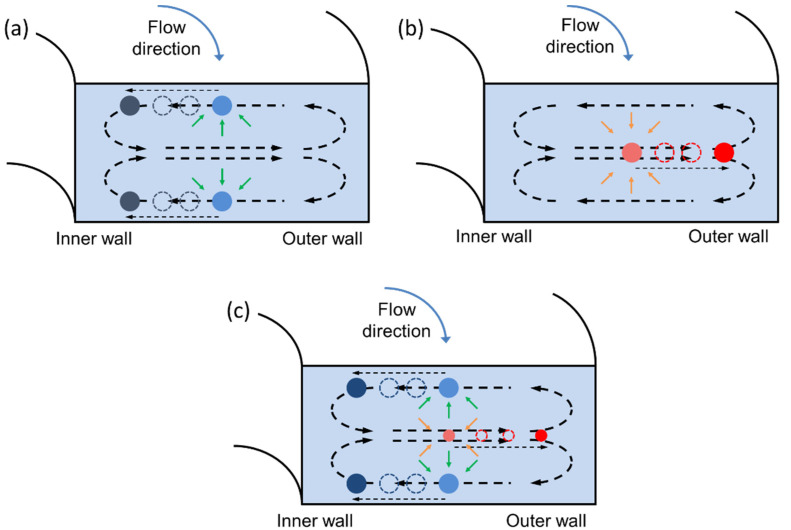
Representation of particle focusing in curved microfluidic channels. (**a**) In Newtonian fluids, particles are driven towards the inner wall. (**b**) In the viscoelastic regime, particles are driven towards the outer wall. (**c**) In the intermediate regime, size-based separations can be achieved.

Inertial microfluidics depends on the flow lift forces that drive suspended particles to focused locations within a confined microfluidic channel. These focused positions arise as basins of attraction [34] where the inertial forces on particles are balanced. In a straight channel with a Newtonian flow, the shear gradient lift force drives particles up the shear gradient towards channel walls. When particles approach the channel wall, the wall repulsive force pushes particles away from the wall. The balance of shear gradient lift force and wall repulsive force form particle focusing positions with zero net forces [35,36]. In a channel with a rectangular cross-section as is shown in Figure 1a, a higher shear stress gradient appears in the channel height direction since the channel height is smaller than the channel width. As a result, particles have two focusing positions in the vertical center of channel top and bottom walls. In the inertial flow, the inertial lift force is determined by the flow velocity gradient and particle dimension. It can be calculated by Equation (1) [35].

*F_L_ = f_L_ρU_max_^2^a^4^/h^2^* (1)

where *f_L_* is the lift coefficient, *ρ* is the fluid density, *U_max_* is the maximum flow velocity, *a* is particle diameter, and *h* is channel height.

In viscoelastic flow, the non-linear shear stress distribution induces particle viscoelastic focusing. This elastic force acts inwards from all of the walls driving the particles towards the channel centerline as shown in Figure 1b. The viscoelastic focusing force is determined by the first normal stress difference and particle dimension. It can be calculated by Equation (2) [37].

*F_E_* = 8*a*^3^*λ*(*U_max_*/*h*)^3^ (2)

where *λ* is the flow relaxation time.

The ratio of *F_E_* and *F_L_* is used to evaluate the dominant force on the particle. With an increase of particle diameter, *F_L_* dominates particle movement. With the increase of *λ* and *U*, *F_E_* dominates particle movement. Based on Equation (3), it is possible to guide large particles to inertial focusing positions and small particles to viscoelastic focusing positions, and the change of particle focusing mechanism leads to a significant change of particle focusing position.

*F_E_/F_L_ = 8λU_max_/f_L_ρah* (3)

In a curved channel, a Dean flow is generated due to the imbalance of pressure and velocity gradient [38]. Dean flow vortices appear in the channel cross-section and flow towards the channel outer sidewall in the channel center and circulate back to the channel inner sidewall near the channel top/bottom sidewall. The influence of Dean flow on particle movement is characterized by the Dean drag force *F_D_* [37]:

(4) $$F_{D}=5.4\times 10^{-4}\pi \mu De^{1.63}a$$

where *De* is the dimensionless Dean number. *F_D_* has lower magnitude when compared with *F_E_* and *F_L_*, while it determines particle focusing position in the horizontal direction when *F_E_* is balanced with *F_L_*. Particle lateral migration in the horizontal direction due to Dean flow is used to increase the distance between particle focusing streams [39]. As a result, an enhanced separation resolution is realized in a curved microchannel.

## 3. Methods

### 3.1. Microfluidic Device Design and Fabrication

**Figure 2:**
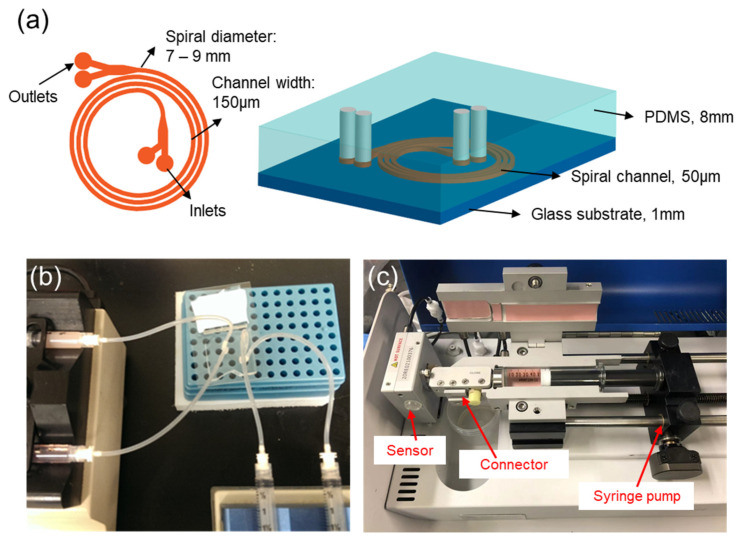
Picture of experimental device set up. (**a**) Schematic view of spiral channel device. (**b**) Microfluidic chip channel connection for particle loading and flow rate control. (**c**) Device structure of m-VROC rheometer.

In this work, we fabricate a device composed of a spiral channel with two inlets and two outlets (Figure 2). Our chip contains a channel with a cross-section of 200 μm × 50 μm (width × height). The channel forms a spiral with a radius that varies between 7–9 mm. There are 3 loops and the total channel length is 172 mm. The channel geometry is fabricated in polydimethylsiloxane (PDMS), according to traditional soft lithography protocols that we have explained previously [39]. Briefly, negative photoresist SU-8 (SU-8 2050, MicroChem, Westborough, MA, USA) is used for the mold fabrication. SU-8 is spin coated on a clean 100 mm (4 inch) wafer, and baked at 95 °C for 15 min. The pattern of the spiral channel is transferred on the photoresist layer using a UV mask aligner, and a post-baking process is performed to stabilize the cross-linked SU-8. Then, the mold is developed using SU-8 developer and the mold quality is checked under a microscope. Uncured PDMS (Sylgard 184, Dow, Midland, MI, USA) is mixed with a curing agent at a ratio of 10:1. Air bubbles generated from the mixing process are removed using a vacuum chamber. About 40 mL degassed PDMS mixture is poured on the SU-8 mold, which leads to a PDMS device with a thickness of 8 mm. PDMS is cured in an oven at 80 °C for 6 h. The cured PDMS is sealed with a glass slide. The PDMS and glass slide surfaces are treated by oxygen plasma to generate permanent bonding. The oxygen plasma treatment is carried out using a Technics PEII-A plasma system, with 200 W power, and 50 sccm O_2_ flow for 2 min. After the bonding process, a post-baking process is performed by putting the spiral channel device on a hotplate at 120 °C for 15 min.

### 3.2. Experimental Set-Up

Two sets of data are collected: one to establish an understanding of fluid elasticity and flow rate and one to explore the application of these findings in detection and separation protocols. The details of the experimental variables are introduced in Table 1. For both sets of experiments, images are acquired of fluorescent beads suspended in the liquid flowing through the channel at a controlled flow rate. Fluorescent beads with diameters of 3 µm(18861-1), 10 µm(19103-2), and 20 µm(19096-2) were purchased from Polysciences, Inc., (Warrington, PA, USA), and beads with diameter of 8µm(FSFR007) were purchased from Bangs Laboratories, Inc., (Fishers, IN, USA). Fluorescent beads are initially suspended in water, and PEO concentrations of 0.001%, 0.003%, 0.005%, 0.025%, 0.05%, 0.1%, 0.2%, and 0.4% by weight are created with serial dilutions of water. The flow rate is controlled with a syringe pump connected to the inlet of the device as is shown in Figure 2. The chip is imaged through the glass near the channel outlet.

### 3.3. Characterization Experiments

Fluorescent beads of 3 and 8 µm are suspended in three different PEO-water dilutions and infused into the spiral channel at seven different flow rates. The bead concentration is kept low (0.1 M/mL) to limit particle–particle interactions. Images are acquired with a high-speed fluorescent camera and about 100 images collected over 1 s are compiled to form the images presented. The spiral channel device is rinsed with water between successive experiments, and experiments with a single chip are acquired starting from the lowest concentrations of PEO to mitigate the possibility of cross-contamination between experiments.

### 3.4. PEO Solution Viscosity Characterization

For viscoelastic aqueous solutions with various PEO concentrations, viscosity is directly associated with peak-to-peak separation distance and resolution. A pressure sensing microfluidics rheometer m-VROC (RheoSense, LLC, San Ramon, CA, USA) is employed in this work that combines a microfluidic channel and a pressure sensor array embedded along the microchannel to measure dynamic viscosity [40]. The principles of VROC are based on measuring the pressure drop by using the Hagen–Poiseuille law, where fluid flows through a given enclosed rectangular slit microfluidic channel. The apparent viscosity depends on the applied shear rate for dilute PEO solutions. The flow rate is proportional to the shear rate; a syringe pump is applied to control the flow rate of an optimal dilute PEO solution. The microfluidics channel etched into a silicon chip contains a depth of 20 um, a width of the 3 mm, and a length of 10 mm. The shallow depth of the microfluidics channel allows one to investigate high shear rates without occurrence of turbulence.

All PEO concentrations from 0.001% to 1% have a measured viscosity at an apparent shear rate between 1.7 × 10^4^ and 1 × 10^5^/s, a constant flow rate from 142.6 µL/min to 823 µL/min through the measuring channel where the pressure sensors monitor the pressure drop at room temperature. The PEO solution is pre-filtered with 0.2 µm PTFE filters and degassed to avoid fibers and bubbles at high shear rate measurements. (The relaxation time is measured by using the Zimm module at 810 µs. Hydrodynamic radius *R_H_* is 68.8 nm, calculated from the Einstein module.)

(5) $$\lambda_{Zimm}=\frac{\eta_{s}\left[\eta \right]M_{w}}{RT}$$

(6) $$R_{H}={\left(\frac{3\left[\eta \right]M_{w}}{10\;\pi N_{A}}\right)}^{1/3}$$

where *η_s_* is the solvent viscosity, [*η*] is PEO intrinsic viscosity, *M_w_* is PEO molecular weight, *R* is the molar gas constant *R* = 8.314 J/(K mol), and *T* is the temperature (K).

### 3.5. Detection and Separation Experiments

Fluorescent beads of 3, 10, and 20 μm are suspended in four different PEO-water dilutions and infused into the spiral channel at three different flow rates using a syringe pump KDS200 (KD Scientific, Holliston, MA, USA). Separation device outlets are connected with another syringe pump (Chemyx F200X, Stafford, TX, USA) for flow withdrawal during sample loading. The bead concentration is much higher (10–100 × 10^6^/mL) to replicate concentrations common to biological samples. Images are acquired with a fluorescent microscope and camera (Nikon A1R). The 4X objective lens provides a field of view of 4 mm × 4 mm to observe the spiral channel outlet region. Fluorescent imaging lasers, including a 405 nm diode laser, a 488 nm Argon gas laser, and a 638 nm diode laser, are used for the observation of fluorescent beads with different colors. Filter cube sets are selected for the optimization with DAPI, FITC, and TRITC signal detection. The spiral channel device is rinsed with water between successive experiments, and experiments with a single chip are acquired starting from the lowest concentrations of PEO to mitigate the possibility of cross-contamination between experiments.

### 3.6. Data Processing

Quantitative data is compiled from images by a Matlab program that reads and interprets the pixel intensity across the channel width. The program averages values across the columns of a selected area, which is manually selected. For comparison and presentation, the data is then reformed to a length of 100 entries, and normalized by the total value of all intensity measurements.

The peak-to-peak separation distance is calculated as a distance between the maximum values of the two distributions. The separation resolution is calculated as:

(7) $$R=\frac{Separation\;Distance}{2\left(W_{1}+W_{2}\right)}$$

where *W*_1_ and *W*_2_ represent the width at half maximum calculated from the distributions [41].

## 4. Results & Discussion

### 4.1. PEO Solution Characterization

**Figure 3:**
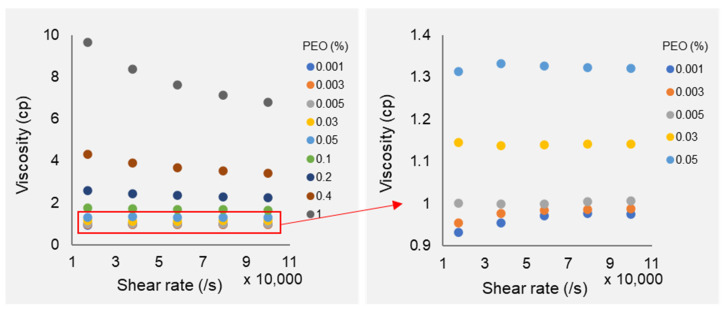
PEO solution viscosity under high shear rates. High concentration PEO solutions are shear-thinning with an increase of flow rate. Low concentration PEO solutions (details are shown on the right figure) exhibit constant shear rate.

Capillary viscometers or cone plate rheometers are commonly used for the precise description of fluid viscosity and viscoelastic behavior. In our application, the shear rate in the microfluidic channel is in the range of 10,000 to 60,000/s, which is much higher than the measurement limit of these measurement methods. The rheometer m-VROC is used to evaluate PEO solution viscoelastic property at a wide range of shear rates. Figure 3 shows the viscosity of the PEO solution with the shear rate of 10,000 to 100,000/s. The ultra-low concentration of the dilute PEO solutions, PEO concentrations of 0.001%, 0.003%, 0.005%, 0.025%, and 0.05% exhibit Newtonian behavior as their viscosities do not significantly change in a lower shear rate range. The viscosity values can be considered as constant. However, higher PEO concentrations greater than 0.05% show non-Newtonian shear-thinning behavior as their viscosities reduce while the shear rate increases. The true wall shear rate is corrected by applying the rigorous Weissenberg–Rabinowitsch correction [42].

(8) $${\dot{\gamma }}_{true}=\frac{{\dot{\gamma }}_{apparent\;}}{3}\;\left(\;2+\frac{d\;ln\;{\dot{\gamma }}_{apparent\;}}{d\;ln\;\tau }\right)$$

where, ${\dot{\gamma }}_{true}$ is the corrected true shear rate, ${\dot{\gamma }}_{apparent\;}$ is the apparent shear rate measured by using m-VROC, and τ  is the wall shear stress in the rectangle slit microchannel.

### 4.2. Particle Movement in Low Concentration PEO Solutions

**Figure 4:**
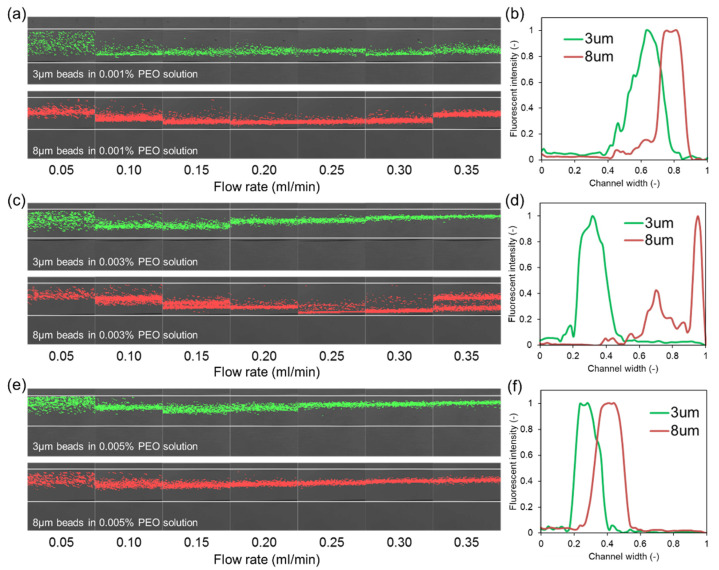
Distribution of 3 μm beads (green) and 8 μm beads (red) near the outlet of the spiral channel device. (**a**,**c**,**e**) represents particle focusing stream under different flow rates in PEO solutions with concentration of 0.001%, 0.003%, and 0.005%. (**b**,**d**,**f**) represent the corresponding particle focusing stream distributions at 0.3 mL/min.

Figure 4 shows the distribution of 3 and 8 µm particles near the outlet of the spiral channel device in low concentrations PEO solutions. As seen from the fluorescent images of particle focusing, the focused positions of particles within the channel width are highly dependent on the concentration of PEO in the fluid. With an increase of PEO concentration, particle streams shift from the inertial focusing regime to the viscoelastic focusing regime. Particles with different sizes have different transitional PEO concentrations, leading to the change of particle separation behavior.

When the concentration of PEO measures 0.001%, the viscoelastic focusing force is low, and the inertial focusing force dominates particle movement as shown in Figure 1a. Both the 3 and 8 µm particles are focused towards the inner side of the channel under all tested flow rates. The diagram of stream distribution indicates that 8 µm particles have a focusing position closer to the channel inner sidewall due to Dean flow. However, the focused peaks of the two particles are very close to each other with significant overlap. This proximity could inhibit the detection of two different types of particles and renders separation impossible.

In comparison, when the concentration of PEO is 0.005%, the viscoelastic force dominates particle movement (Figure 1b). Both the 3 and 8 µm particles are focused towards the outer side of the channel, and the 3 µm particles have focusing positions closer to the sidewall. The focused bands are tightest at 0.30 mL/min, representing approximately 10% of the channel width for both particle types. As shown in the right column of Figure 4, the focused peaks of the two particles are still very close to each other with significantly overlapping peaks. Again, this proximity inhibits the detection of two different types of particles and would make complete separation impossible. The focusing stream width in the viscoelastic focusing regime is smaller than that in the inertial focusing regime. It could be explained by the fact that particles have two focusing streams in the vertical direction in inertial focusing, while there is only one focusing stream in the viscoelastic focusing.

Between the two concentrations, when the concentration of PEO is 0.003%, particles with different sizes are dominated by different focusing regimes as shown in Figure 1c. The movement of 3 µm particles is dominated by the viscoelastic focusing. Particles get focused to the channel centerline and pushed to the outer sidewall by Dean flow. The movement of 8 µm particles is dominated by inertial focusing. Particles become focused to the channel top and bottom wall and are pushed to the inner sidewall by Dean flow. With the beads focused towards different edges of the spiral channel, we observe the complete separation of the two focused peaks which are also spread by about half of the channel’s width. This greatly enhanced separation is beneficial for both detection and separation as will be explored in the following section.

Compared with the particle dimension or PEO concentration, the flow rate does not have a significant influence on the particle focusing regime. The increase of flow rate promotes inertial focusing and viscoelastic focusing and leads to the increase of the Dean flow velocity. Particle focusing stream width decreases with the increase of flow rate and the tightest focusing stream occurs at a flow rate between 0.20 and 0.30 mL/min for all cases. In addition, it is observed that particle focusing position is close to the channel sidewall since the Dean flow velocity increases. At a flow rate of 0.35 mL/min, particles dominated by the inertial focusing regime (3 and 8 µm particles in 0.001% PEO, 8 µm particles in 0.003% PEO) have focusing streams shift towards the channel center. In this case, the increase of flow rate leads to the increase of viscoelastic force, and particle focusing positions change.

### 4.3. Particle Movement in Medium Concentration PEO Solution

**Figure 5:**
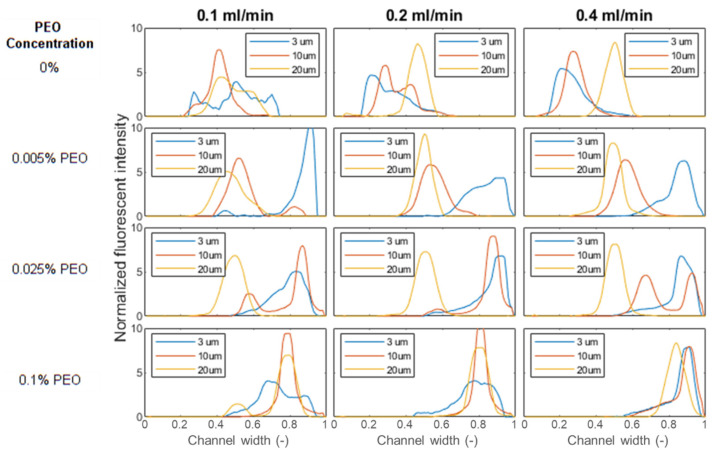
Plots showing focusing of 3, 10, and 20 μm beads under varied flow conditions. Across the rows of the figure, the flow rate increases from left to right while the PEO concentration is increased down each of the rows. The x-axis shown in each figure is the normalized channel width from channel inner side wall to outer side wall.

With the increase of PEO concentration, the viscoelastic force increases, and the balance between *F_E_*, *F_L_* and *F_D_* need to be configured. Small-sized particles have viscoelastic dominated movement in low concentration PEO solution, while large-sized particles experience a high inertial effect even in medium concentration PEO solution. In the experiment, focusing behaviors of 3, 10, and 20 µm particles at flow rates of 0.1, 0.2, and 0.4 mL/min are observed in three different PEO concentrations. Plots of the data set are shown in Figure 5. In Figure 5 we can observe many of the same trends that were present in our initial data set. At the lowest PEO concentration, particles of all sizes are focused to the inner side of the channel, with the tightest focusing achieved by larger particles and focusing improving at the higher flow rates. Particle focusing position is highly affected by particle dimension and PEO concentration. 3 µm beads have a focusing position close to the outer sidewall in all PEO solutions. 10 µm beads focus to the channel center in 0.005% PEO solution, and the focusing position migrates to the channel outer sidewall in 0.025% PEO solution. 20 µm beads changed focusing position in 0.1% PEO solution.

**Figure 6:**
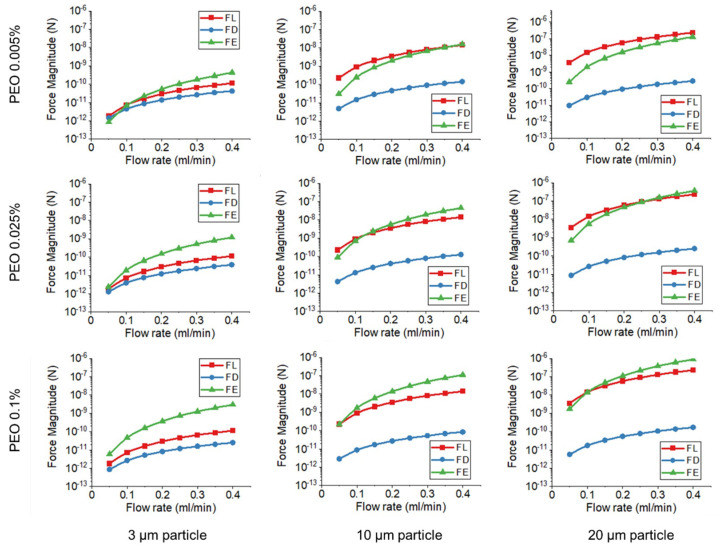
Force magnitude of *F_L_*, *F_E_*, and *F_D_* in medium concentration PEO flow.

The increase of PEO concentration leads to the increase of *F_E_*, and viscoelastic force bears a high influence on particle movement. With the increase of particle size, *F_L_* grows faster than *F_E_*, and the inertial effect dominates particle movement. The balance between *F_E_* and *F_L_* determines particle focusing position. Figure 6 shows the magnitude of *F_L_*, *F_E_*, and *F_D_* in the current data set. All forces increase with flow rate, while F_E_ has a higher increase rate compared with *F_L_*. It should be noticed that *F_D_* has a much low magnitude compared with *F_E_* and *F_L_* for large-sized particles, and the effect of *F_D_* decreases with the increase of PEO concentration.

**Figure 7:**
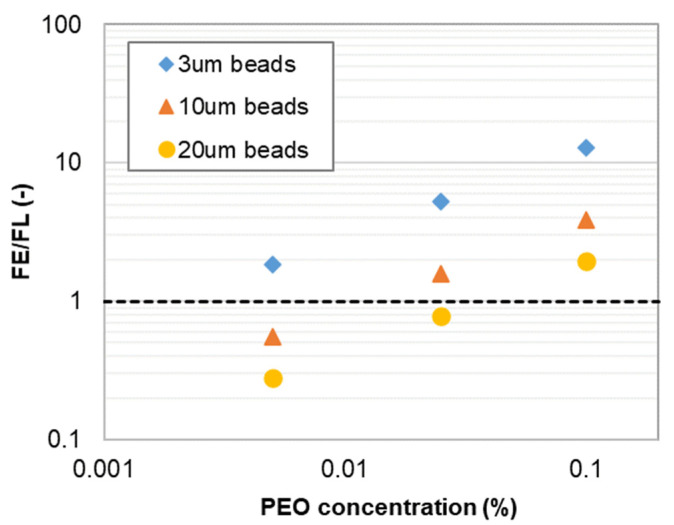
The ratio of *F_E_/F_L_* under different PEO concentrations at 0.2 mL/min.

For large-sized particles, a focusing position in the channel center is observed when *F_L_* > *F_E_*. Large-sized particles occupy a large volume in the confined channel. 3, 10, and 20 µm particles have particle to channel height ratios *a/h* of 0.06, 0.2, and 0.4, respectively. When *F_L_* dominates large particle movement, particles tend to migrate towards the channel top/bottom wall, while this movement is balanced by the wall repulsive force. As a result, particles have focusing positions in the middle between the channel top/bottom wall and channel center. At such a focused position, the Dean flow is counter-balanced on the particle, and will not drive particle towards the channel sidewalls in the horizontal direction. Particles have focused positions in the channel center. Figure 7 illustrates the ratio of *F_E_* and *F_L_* in different PEO concentrations, which can be used to explain particle movement behavior. For 3 μm beads, *F_E_*/*F_L_* is above one in all three PEO concentrations. *F_E_* dominates particle movement and 3 μm beads have a focus position near the channel outer sidewall. For 10 μm beads, *F_E_/F_L_* is above one in 0.025% PEO solution, and the transition of focusing positions occurs. In this case, *F_E_/F_L_* = 1.58 in 0.025% PEO solution. The magnitudes of F_E_ and *F_L_* are very close to each other, leading to the double peak focusing phenomenon since particles may have stable focusing positions under two focusing regimes. For 20 μm beads, the particle changed focusing position in 0.1% PEO solution. It should be noticed that *F_E_/F_L_* = 0.28 in 0.005% PEO, and the inertial force dominates 20 μm bead movement. However, the 20 μm beads are notably large compared with the channel height (50 μm), the inertial focusing position is close to the channel center, and Dean flow is still counter-balanced in this case.

### 4.4. Particle Movement in High Concentration PEO Solution

**Figure 8:**
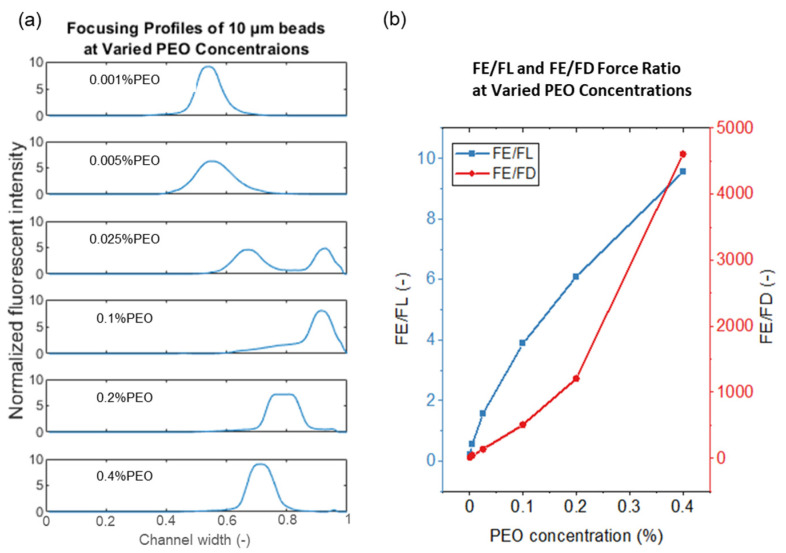
(**a**) Effect of PEO concentration on the focusing of 10 μm beads at a constant flow rate of 0.2 mL/min. The PEO increases in each figure from top to bottom with the absolute value shown in the legend. The x-axis shows the normalized channel width from the channel inner side wall to the outer side wall. (**b**) Force ratio of F*_E_/F_L_* and *F_E_/F_D_* for 10 μm beads.

When the PEO concentration increases above 0.1%, the PEO solution is a semi-dilute solution, in which the polymer molecules start to interact with each other, leading to a change of polymer solution properties. In a semi-dilute PEO solution, a viscoelastic secondary flow appears in the channel cross-section, leading to a multiple stream focusing (MSF) phenomenon. The mechanism and application of MSF in high viscoelastic flow is described in detail in our previous publication [43]. MSF is induced by a viscoelastic secondary flow drag force and exhibits a larger influence on small-sized particles. Large-sized particles are less affected by MSF and maintain a single focusing stream, while the focusing stream position changes in high concentration PEO solution. Figure 8a shows the 10 µm particle focusing stream position in different PEO solutions. It is found that the focusing stream moves from the channel outer sidewall towards the channel center in high concentration PEO solutions. With the continuous increase of PEO concentration, the solution viscosity and relaxation time increase dramatically, and the flow is purely viscoelastic-dominated. Particles are focused to the channel center due to the viscoelastic force and are driven by Dean flow to the channel outer sidewall. With the increase of PEO concentration, the Dean flow has less effect on particle focusing positions. Figure 8b shows the ratio of *F_E_* to *F_D_* in high concentration PEO solution. The increase of solution viscosity reduces the Dean flow drag force, and *F_E_/F_D_* increases from 500 to 4500 in high concentration PEO solution. With the combination of these factors, particle movement is less affected by the Dean force, and the focusing position shift towards the channel centerline.

### 4.5. Detection Resolution

**Figure 9:**
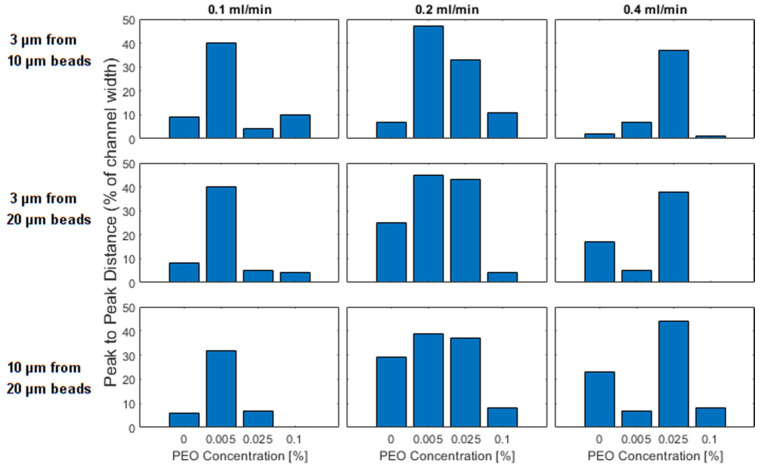
Peak to peak distance as a percentage of the channel width) between the peaks formed in the focusing of differently sized particles. In the first row, 3 and 10 μm beads are compared, in the second row 3 and 20 μm beads are compared and in the third row 10 and 20 μm beads are compared. The columns represent the flow rates of 0.1, 0.2, and 0.4 mL/min. The individual bars within a plot represent different PEO concentrations which increase from left to right.

As discussed previously, size-based separations of particles were achieved utilizing a spiral channel to separate beads suspended in a Newtonian fluid. However, since both of the focused peaks will occur within the inner half of the channel, there is a limit to the distance that peaks can be focused, and in our results, even in the case of the largest separation (3 μm from 20 μm beads at 0.2 mL/min), this separation is limited to less than one third of the channel width. However, by utilizing viscoelastic fluids we can selectively shift the peak of the smaller particle to drive it to the far outside of the channel. This allows us to create an even greater distance between the peaks of the two different particles. In Figure 9 we report the separation that we observe in the 9 tested configurations. The most dramatic improvement is in the case of 3 μm and 10 μm beads which have focused peaks within one tenth of the channel width in Newtonian fluid at all flow rates, but which can be separated by >40% of the channel width at 0.005% PEO at 0.2 mL/min.

For all combinations of bead sizes and flow rates, the best separation is identified in one of the two intermediate PEO concentrations. There is no separation that cannot be improved by adding a small concentration of PEO into the fluid. In preparing to apply these results, it is important to remember that the distances are reported without regard for the location, i.e., in the Newtonian regime 3 μm beads will be focused further inside while they will later be focused further to the outside of the channel.

The ability to increase the separation distance between focused peaks could prove helpful in separation and detection applications where the signal of similarly sized particles may otherwise overlap. More generally, especially in the transition region, since the focusing behavior represents such a strong function of flow rate, PEO concentration, and bead size, we imagine that the proposed spiral channel device could be employed as a sensor for any of the other variables if two of the values were known.

**Figure 10:**
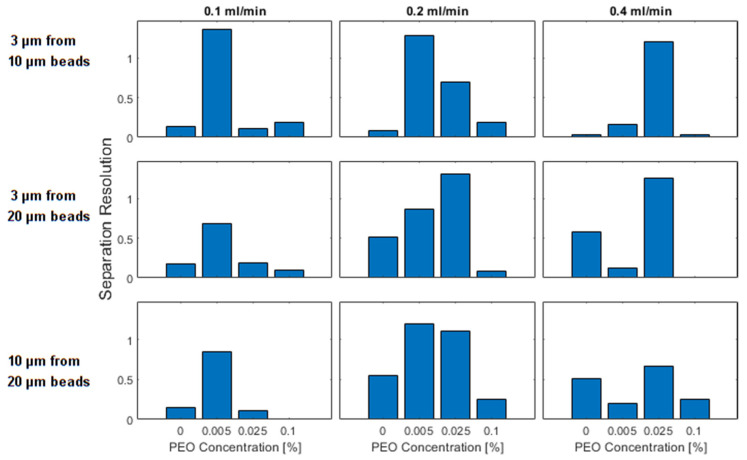
Separation resolution as a percentage of the channel width between the peaks formed in the focusing of differently sized particles. In the first row, 3 and 10 μm beads are compared, in the second row 3 and 20 μm beads are compared and in the third row 10 and 20 μm beads are compared. The columns represent the flow rates of 0.1, 0.2, and 0.4 mL/min. The individual bars within a plot represent different PEO concentrations which increase from left to right.

Increased separation between focused peaks is also an indicator that increased particle separation should be achievable; we demonstrate that with the aid of an additional performance metric, separation resolution, which attempts to quantify our ability to create separation between two focused peaks. Again, the performance metrics of all 9 operating conditions is presented in Figure 10 with many trends consistent with the plots of the distance between focused peaks. All separations can be improved with the introduction of low quantities of PEO, either at 0.005% or 0.025% PEO.

## 5. Conclusions

In this study, particle focusing position in PEO solutions with a wide range of concentrations was studied experimentally. The achieved results are used for the analysis of the particle focusing regime and improvement of separation resolution. PEO solution viscoelastic properties in high shear rate flow are characterized, and the variation of solution viscosity under different shear rates affects particle movement in the viscoelastic flow. Particles with different sizes bear different focusing positions with the increase of PEO concentration and flow rate, and the particle focusing process is determined by the balance of inertial flow, viscoelastic flow, and Dean flow. Therefore, by precisely manipulating the PEO concentration of the fluid, we can create a situation in which smaller particles are driven to the outer side of the channel while larger beads are driven to the inside of the channel. This condition allows for an increased separation distance between their focused peaks, which could assist with the detection of beads of different sizes, and an increased separation resolution which could help in separation protocols.

## Figures and Tables

**Table 1:** Summary of experiment parameters in the spiral channel particle separation test.

Variables	Value
Channel geometry	150 µm width; 50 µm height; 172 mm length
PEO concentration	0.001%, 0.003%, 0.005%, 0.025%, 0.05%, 0.1%, 0.2%, and 0.4%
Particle diameters	3 µm, 8 µm, 10 µm, and 20 µm
Flow rate	0.05 mL/min, 0.1 mL/min, 0.15 mL/min, 0.2 mL/min, 0.25 mL/min, 0.3 mL/min, and 0.35 mL/min

## Data Availability

The data that support the findings of this study are available from the corresponding author, B.G, upon reasonable request.
